# Role of Novel Retroviruses in Chronic Liver Disease: Assessing the Link of Human Betaretrovirus with Primary Biliary Cirrhosis

**DOI:** 10.1007/s11908-014-0460-7

**Published:** 2015-03-10

**Authors:** David Sharon, Andrew L. Mason

**Affiliations:** 1 Department of Medicine, University of Alberta, Edmonton, AB Canada; 2 Division of Gastroenterology and Hepatology, Center of Excellence in Gastrointestinal Inflammation and Immunity Research, University of Alberta, 7-142 KGR, Edmonton, AB T6G 2E1 Canada

**Keywords:** Autoimmune liver disease, Primary biliary cirrhosis, Human betaretrovirus, Mouse mammary tumor virus, Viral pathogenesis, Combination antiretroviral therapy

## Abstract

A human betaretrovirus resembling mouse mammary tumor virus has been characterized in patients with primary biliary cirrhosis. The agent triggers a disease-specific phenotype in vitro with aberrant cell-surface expression of mitochondrial antigens. The presentation of a usually sequestered self-protein is thought to lead to the loss of tolerance and the production of anti-mitochondrial antibodies associated with the disease. Similar observations have been made in mouse models, where mouse mammary tumor virus infection has been linked with the development of cholangitis and production of anti-mitochondrial antibodies. The use of combination antiretroviral therapy has been shown to impact on histological and biochemical disease in mouse models of autoimmune biliary disease and in clinical trials of patients with primary biliary cirrhosis. However, the HIV protease inhibitors are not well tolerated in patients with primary biliary cirrhosis, and more efficacious regimens will be required to clearly link reduction of viral load with improvement of cholangitis.

## Introduction

Primary biliary cirrhosis (PBC) is a complex autoimmune liver disease of unknown etiology [1]. Our team first characterized a human betaretrovirus resembling mouse mammary tumor virus (MMTV) in PBC in 2003 [2]. Since then, our research has been directed towards creating diagnostic virological assays, assessing the role of MMTV in mouse models of autoimmune biliary disease and conducting clinical trials using antiretroviral therapy. Attention has also been directed towards genome-wide association studies in populations with PBC [3–6]. Collectively, these studies show that much of the genetic risk associated with PBC is linked with the pathogenesis of other immune disorders. Furthermore, it has been theorized that several polymorphisms linked with PBC and related immune disorders confer a relative state of immunodeficiency, suggesting diseases may arise because of an inability to combat specific microbial infections [7, 8]. The shift in emphasis towards inadequate immune responses has also been seen in many of the “spontaneous” immunodeficiency mouse models for PBC that will be discussed in more detail in this review [7, 9]. A parallel of autoimmune phenomena occurring in the setting of immunodeficiency has also been documented in patients with HIV infection. In this setting, autoantibody production occurs in parallel with progressive lymphopenia and immune dysregulation [10]. Also, the pathological features of opportunistic infections often resemble a phenotype similar to autoimmune liver disease, such as AIDS-related cholangiopathy resembling sclerosing cholangitis [11]. Herein, we discuss the circumstantial data linking a human betaretrovirus with PBC and provide direction for further studies that may bridge the gap for providing robust data supporting a causal relationship for microbe and disease.

## Primary Biliary Cirrhosis

PBC is an autoimmune cholestatic liver disease associated with the progressive immune destruction of intrahepatic bile ducts and production of anti-mitochondrial antibodies (AMA) [1]. It is a rare disease occurring in 1:2500 to 1:100,000 worldwide with an increasing prevalence moving away from the equator. PBC is 10 times less common in men and observed in approximately 1 in 500 middle-aged women [1]. The histological disease is characterized by a non-suppurative cholangitis with granulomatous destruction of 30- to 80-μm interlobular bile ducts. The progressive ductopenia leads to the accumulation of bile in the liver resulting in fibrosis. Patients present with fatigue, itching, sicca syndrome, and occasionally right upper quadrant pain. To make the diagnosis of PBC, patients require two of three of the following criteria with cholestatic liver tests, AMA serology, and/or liver histology compatible with PBC [1].

The only licensed therapy for PBC is ursodeoxycholic acid (UDCA) therapy, which acts as a choleretic agent to eliminate bile from the liver. However, a third of patients still develop progressive disease, and as a result, PBC accounts for 10 % of patients requiring liver transplantation in Canada [1]. Apart from the study of ursodiol, clinical trials for PBC have been mainly geared towards investigating immunosuppressive agents. This is because immunosuppression has proven life saving for patients with autoimmune hepatitis. However, the outcomes of similar clinical studies in PBC have been disappointing. Individual treatments have had little impact on halting the progression of PBC, and specific immunosuppressive agents have not, therefore, been adopted because of toxicity or lack of efficacy.

Approximately 95 % of patients with PBC develop AMA targeting the lipoyl group on a member of the pyruvate dehydrogenase complex (PDC)-E2 [12–14]. Interestingly, PDC-E2-like proteins localize to the surface of biliary epithelial cells in PBC patient samples both in vivo and in vitro as well as draining peri-hepatic lymph nodes [15–17]. Of note, autoreactive CD4+ and CD8+ T cells from PBC patients target the same B cell autoantigen, namely the inner and outer lipoyl domains of PDC-E2 [18]. The resultant immune response results in granulomatous destruction of bile ducts, which is thought to occur through recruitment by cytokines upon the activation of toll-like receptors (TLR)-3 and TLR-4 in biliary epithelial cells [19, 20]. However, the role that autoimmunity plays in disease remains to be resolved. AMA levels have little bearing on the disease process; AMA-negative patients have the same prognosis as those with anti-mitochondrial antibodies, and immunosuppression is of limited benefit and only reserved for a subset of patients [2]. However, there is a general agreement that the cell-surface expression of mitochondrial proteins in the setting of an active immune response results in loss of tolerance to PDC-E2 [21].

Family studies suggest that both genetic and environmental factors are implicated in the development of PBC. With regard to an infectious process, unrelated family members can develop disease and PBC clusters geographically in regions, and migration studies show that the children develop the relative incidence of PBC in their adopted host country [22–26]. An infectious disease process is also compatible with observations from liver transplantation. Histological evidence for recurrent PBC is observed in up to 40 % of patients, and more potent immunosuppressive regimens with tacrolimus accelerate the onset and severity of recurrent disease [27–31]. In contrast, cyclosporine A appears to be protective against the development of recurrent PBC, and as a cyclophylin A inhibitor, it has antiviral activity against betaretroviruses, HIV, and other viral agents [31–33]. Bacteria, viruses, and xenobiotics have all been implicated as environmental triggers, and each has been linked in mouse models of disease. However, data is lacking to firmly link any of these triggers in the development of PBC [1]. Only the betaretrovirus has been detected in bile ducts of PBC patients, but these data have not been reproduced by others.

The pathophysiology of PBC is directly related to the loss of bile ducts and accumulation of bile acids within the liver [2]. Accordingly, secondary bile salt, UDCA, is used as a choleretic to help remove toxic bile [34, 35]. Indeed, there are data to suggest that PBC patients’ bile may lack protective factors to counteract the bile acids. For example, PBC patients’ biliary epithelial cells have increased miR-506 expression that blocks the translation of anion exchanger 2 protein, which in turn decreases bicarbonate secretion [36–39]. UDCA therapy can partially restore the protective effect of the bicarbonate “umbrella” to protect against the damage caused by the acidic bile. UDCA generally ameliorates liver disease in most PBC patients and has reduced the frequency for transplantation related to PBC in countries where UDCA treatment is the standard of care [2]. Nevertheless, approximately a third of patients do not respond adequately to UDCA, and there is a need for novel therapeutic approaches to halt PBC.

## MMTV and Breast Cancer

MMTV is endemic in several laboratory mouse strains, where the virus is transmitted exogenously from mother to weanling pups through milk [40]. The milk-associated particle travels from the gut to the spleen where it infects B cells, T cells, and dendritic cells. B cells infected with MMTV present the viral-encoded superantigen, which induces the proliferation of T cells. These, in turn, promote B cell proliferation, leading to enhanced MMTV replication and spread. MMTV has also been shown to promote B cell activation through binding to the TLR-4 receptor [41, 42]. Interestingly, after the B cell bound superantigen stimulates T lymphocyte replication through binding to the Vβ chain of TCR, the T cells eventually die off [43]. Accordingly, MMTV replication is associated with an increased subset of Vβ-restricted cells in virally infected tissues as well as a reduced subset of Vβ-restricted cells in peripheral blood.

During infection, circulating lymphocytes carrying MMTV are passaged to lymphoid tissue, the brain, the liver, and mammary epithelial cells, which deliver multiple viral particles into milk [44]. MMTV is a slow oncogenic virus that may cause breast cancer through a variety of mechanisms. The virus is thought to induce breast cancer mostly through insertional mutagenesis, where MMTV integrates upstream and increases the expression of proto-oncogenes [45]. In addition, MMTV encodes transforming factors both in the Gag protein and Env protein that contains an immunoreceptor tyrosine activation motif [46, 47].

MMTV also exists as an endogenous virus passed on from generation to generation due to prior integration into the host germline DNA [40]. In the C3H breast cancer-prone mouse, for example, endogenous proviral MMTV can express exogenous virus that results in the development of disease. However, in most mouse strains, the majority of endogenous proviral sequences are not infectious due to epigenetic factors, mutations and deletions within the genome. Similar to exogenous virus, endogenous MMTV strains encode superantigens that induce the deletion of specific T cells in utero and therefore prevent infection with exogenous strains that are restricted to replicating in the deleted subset of T lymphocytes [43]. Indeed, mice have developed multiple innate and adaptive mechanisms to combat MMTV infection. For example, mice that develop robust IL-12/interferon-γ responses limit the spread of MMTV infection by production of neutralizing Env antibodies [48]. This observation may be relevant to the development of PBC because multiple candidate genes associated with disease encode variants within the IL-12 signaling pathway [3–6].

## Does MMTV Infect Humans?

Interest in whether MMTV could infect humans emerged more than 40 years ago with the detection of betaretrovirus particles by electron microscopy in milk from breast cancer patients [49]. Subsequently, viral proteins and nucleic acid from MMTV were identified in human breast cancer and non-malignant breast tissues, and evidence for immune responses to betaretrovirus proteins also emerged. However, most studies proved difficult to replicate. The virus appeared at the limits of detection, and concerns were raised about whether human endogenous retroviruses were being confused with MMTV. In the 1990s, the “human mammary tumor virus” was cloned and detected in a proportion of breast cancer patient samples by PCR [50]; some groups were able to replicate the studies while others could not. Accordingly, the field has not advanced due to an inability to demonstrate definitive proof of infection, such as the demonstration of proviral integration sites in patients [49].

Some of the obstacles against establishing the presence MMTV-like infection in humans have been resolved however. For example, Beatrice Pogo’s group has isolated MMTV-like particles from primary cultures of human breast cancer cells [51]. Furthermore, it was originally thought that humans could not be infected by MMTV because the human transferrin was unable to act as an MMTV receptor, whereas the mouse transferrin receptor could [52–54]. Since then, in vitro studies have shown that MMTV can infect and replicate in human cells, suggesting that other receptors may be expressed on these cells to allow the entry of MMTV [55–57]. Also, our group has identified a human betaretrovirus genetically indistinguishable from MMTV in biliary epithelial cells and peri-hepatic lymph nodes of PBC patient samples [2, 58].

## Betaretrovirus Infection as a Potential Risk Factor of PBC

The link of retroviral infection with PBC first emerged 15 years ago, when false positivity to HIV p24 and a retrovirus isolated from patients with Sjörgen’s syndrome was discovered in a subgroup of patients [59]. These data were interpreted as serological reactivity to an undefined virus. Subsequently, the human betaretrovirus was cloned from a biliary epithelium cDNA library using a non-biased cloning method; the full-length proviral sequence was then derived from a PBC patient’s peri-hepatic lymph node [2, 58]. Similar to observations in mice, we found that human betaretrovirus levels were higher in peri-hepatic lymph nodes than the liver; three quarters of patients had detectable betaretrovirus RNA in lymph node samples but only a third within the liver. The lack of detectable viral DNA in control subjects was a reassurance against the sequences representing a false-positive detection of a human endogenous retrovirus [60, 61].

Taken together, our studies suggested that prior exposure to MMTV-like agent might have a role in the development of PBC. Indeed, the sequence homology of the human betaretrovirus and MMTV suggested a zoonosis from mice to humans, as previously observed with the human mammary tumor virus sequences found in breast cancer patients. Similar to studies in patients with breast cancer, the human betaretrovirus was at the limits of detection, and therefore, we required the use of a nested PCR to detect viral DNA. As a result, others were unable to replicate our findings. A North American lab could not find any evidence of hepatic betaretrovirus using a single round of PCR [62], whereas a second study from Australia identified betaretrovirus sequences in a small number of liver samples but without any disease specificity for PBC [63]. Collectively, these studies questioned the relevance of betaretrovirus infection in PBC patients, as the virus could not be detected within the liver. Of note, betaretrovirus infection in peri-hepatic lymph nodes was not investigated by other laboratories. Furthermore, there were several inherent problems with PCR studies, not the least of which was that they may lack sensitivity and the ever-present concern with PCR carryover causing contamination.

The isolation of virus from the site of disease and detection of proviral integration sites in the human genome are considered a gold standard for demonstrating retroviral infection. Accordingly, we embarked on using this virological approach to linking human betaretrovirus infection with PBC. In the first instance, PBC patients’ peri-hepatic lymph nodes were co-cultured with Hs578T cells to amplify and isolate the virus. Furthermore, we investigated the presence of betaretrovirus integrations in total DNA extracted from the liver, lymph nodes, and biliary epithelium cells extracted from liver transplant recipients. Using ligation-mediated PCR and next-generation sequencing, we obtained more than 1500 novel integration sites from PBC patients, as well as patients with autoimmune hepatitis, but rarely in control subjects. Of interest, betaretroviral integrations were seldom detected in whole liver DNA but were regularly identified in the majority of PBC patients’ biliary epithelium and lymph nodes [64]. While these studies provided firm evidence for infection at the site of disease, it remains to be determined how a barely detectable viral load can be associated with disease. Moreover, these data need to be reproduced by others to gain traction in the scientific community. Finally, the findings merely suggest a link of infection with disease, and additional studies are clearly required to provide evidence for a causal association of betaretrovirus infection and PBC.

## Betaretrovirus Infection Triggers the PBC-Specific Phenotype

The characterization of a human betaretrovirus in patients with PBC 10 years ago was unexpected because most investigators were working on the hypothesis that bacteria triggered autoimmunity by the process of microbial molecular mimicry. Indeed, anti-mitochondrial antibodies have been shown to react with many bacteria that share antigenic determinants with human PDC-E2 and other highly conserved oxo-acid dehydrogenase proteins. In case-control studies, however, no bacterial candidate has been convincingly found in PBC patients’ liver or bile duct samples [21].

In contrast, betaretroviruses have been shown to trigger aberrant PDC-E2 expression suggesting a straightforward infectious disease model for the generation of loss of tolerance to self [21]. In preliminary studies, co-cultivation of homogenized lymph nodes from PBC patients with biliary epithelium resulted in an increased expression of PDC-E2-like antigens reactive to AMA on the cell surface, whereas control lymph nodes had no such effect [65]. Follow-up studies showed that the transmissible agent could be passaged in serial culture and the effect was abrogated by γ-irradiation. This agent was subsequently characterized as a human betaretrovirus resembling MMTV. Pure isolates of MMTV and the human betaretrovirus were shown to trigger the increased AMA reactivity to PDC-E2 in normal biliary epithelial cells [2, 64]. Notably, the in vitro cell culture model neatly paralleled in vivo observations in PBC patients and mouse models. In studies using PBC patients’ peri-hepatic lymph nodes, for example, cells with demonstrable betaretrovirus proteins also displayed aberrant AMA reactivity [2]. Similarly in mouse models of autoimmune biliary disease with spontaneous AMA production, MMTV proteins and increased PDC-E2 cell-surface expression were observed in lymphoid tissues and biliary epithelium [9].

## MMTV and Cholangitis Mouse Models

Specific criteria loosely based on Koch’s postulates have been proposed to demonstrate the central role of specific antigens in autoimmune disease [66]. These Witebsky’s postulates propose that an autoimmune disease ought to be reproduced in animals challenged with the disease-specific autoantigen co-administered with adjuvant. However, the first attempts to create a PBC mouse model by administering combinations of PDC-E2 with adjuvant to healthy strains of mice failed to trigger anti-mitochondrial antibody production and biliary disease [67–69]. More recently, several mouse models of autoimmune biliary disease have been described that spontaneously produce anti-mitochondrial antibodies and develop liver disease [70]. It is significant that most of these models are immune deficient and some succumb as a result of diffuse inflammatory disease [7].

The model most studied for MMTV involvement in the generation of autoimmune biliary disease is the NOD.c3c4 congenic mouse [9, 71]. This line was originally modified to identify genes involved in type-1 diabetes in the non-obese diabetic (NOD) mouse [72, 73]. While NOD.c3c4 mice were protected against the development of diabetes, they developed granulomatous cholangitis, biliary cysts (atypical for PBC), as well as anti-mitochondrial and antinuclear antibodies. Similar to observations in the parental NOD strain, the disease could be transferred to healthy NOD.c3c4-*scid* mice through injection of splenocytes, as well as isolated CD4+ T cells, from diseased mice [74]. Interestingly, the NOD.c3c4.Igμ−/− mice lacking B-lymphocytes were found to have reduced inflammatory disease and cholangitis [75]. These studies demonstrate an essential role for both T- and B-lymphocytes in the development of autoimmune biliary disease in this model. Other immune-deficient models were also described that develop spontaneous anti-mitochondrial antibody production and succumbed from multi-organ inflammation, such as the T cell TGF-β receptor II dominant-negative mouse [76], the IL-2 receptor α-deficient mouse [77], and the Scurfy mouse lacking T regulatory cells [78].

Since MMTV is prevalent in laboratory mice, our group tested the hypothesis that the anti-mitochondrial antibody production occurred as a result of MMTV expression being linked with aberrant expression of PDC-E2 in the NOD.c3c4, the NOD parental strain, the T cell TGF-β receptor II dominant-negative mouse, and the IL-2 receptor α-deficient mouse, using appropriate controls [9]. It is well established that endogenous retroviruses can recombine in mice with specific immune defects to mediate disease [79]. Whereas healthy C57BL/6 mice encode three full-length endogenous MMTV proviruses within the genome but do not express infectious virus. While our studies showed that the C57BL/6 control mice displayed little evidence of MMTV, we noted high expression of MMTV RNA and proteins in the T-cell TGF-β receptor II dominant-negative mice and the IL-2 receptor α-deficient mice, both of which were derived on the C57BL/6 background. Similarly, both the NOD.c3c4 and NOD parental strain demonstrated high MMTV levels. Furthermore, all the AMA-producing mice also had evidence of aberrant expression of PDC-E2 in cells expressing MMTV proteins, such as lymphoid tissues and bile ducts [9].

We next tested whether MMTV inhibition would have an effect on cholangitis development in the NOD.c3c4 model using antiretroviral therapy. Eight-week-old mice were treated for 12 weeks with lamivudine and zidovudine (AZT/3TC) or tenofovir and emtricitabine (TDF/FTC), with or without lopinavir boosted with ritonavir (LPR/r). Response to therapy was determined biochemically and histologically. A pronounced reduction in cholangitis was observed in mice treated with TDF/FTC and LPR/r in comparison to the other groups, including AZT/3TC and LPR/r. Interestingly, a proportion of NOD.c3c4 mice treated with AZT/3TC developed markedly elevated levels of MMTV in the liver, suggesting viral resistance to therapy [71]. Subsequently, mutational analyses of the MMTV *pol* gene showed variants (M188V) similar to those observed with lamivudine resistance in patients with HBV and HIV infection. Taken together, the studies suggest the possibility that the NOD.c3c4 mouse model of autoimmune biliary disease may also be an infectious disease model.

## Clinical Experience with Antiretroviral Therapy in Patients with PBC

**Fig. 1:**
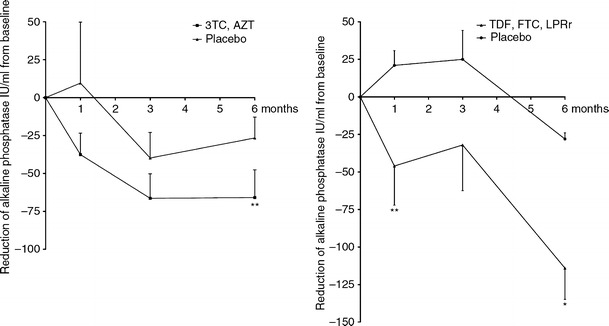
Incremental improvement of hepatic biochemistry observed in PBC patients maintained on UDCA receiving combination antiretroviral therapy with a protease inhibitor. Patients treated with daily lamivudine 150 mg (3TC) and zidovudine 300 mg (AZT) developed a 66 IU/mL mean reduction in ALP, whereas those receiving daily tenofovir/emtricitabine 300/200 mg (TDF, FTC) and lopinavir/ritonavir 800/200 mg (LPRr) for 6 months (*n* = 13) experienced a mean ALP reduction of 114 IU/mL [two-way ANOVA, *P < 0.001, **P < 0.05; adapted from ref. 84 with permission]

Several clinical trials have been conducted to assess whether inhibition of betaretrovirus infection can impact on the disease process in patients with PBC. In open-label studies, PBC patients on maintenance ursodeoxycholic acid therapy received treatment with the reverse transcriptase inhibitors, 3TC, or combination AZT/3TC for 12 months [80]. The study showed that while 3TC had little effect on liver damage, AZT/3TC had an impact both biochemically and histologically with improvement in bile duct injury, ductopenia, and necroinflammatory score. The return of bile ducts is important as no other therapy has reversed ductopenia in PBC patients [80]. Of interest, biochemical breakthrough occurred with both lamivudine and AZT/3TC therapy consistent with observations of *pol* mutations in the mouse model [81]. Subsequent randomized control study of AZT/3TC therapy for PBC patients was disappointing as patients on therapy did not achieve the stringent endpoints of normalizing alkaline phosphatase levels [82]. Nevertheless, AZT/3TC treatment was associated with significant improvements in hepatic biochemistry (Fig. 1).

Combination antiretroviral therapy with TDF/FTC and LPR/r has shown efficacy in the NOD.c3c4 mouse model and was successfully used in clinical practice to normalize hepatic biochemistry in a newly diagnosed PBC patient co-infected with HIV and human betaretrovirus [83]. We have also treated a young PBC patient with severe recurrent disease following liver transplantation with TDF/FTC and LPR/r. The patient had an excellent clinical and biochemical response, and two repeat liver biopsies in 2013 and 2014 showed diminished histological disease. With this knowledge, we embarked on a randomized controlled trial to test the efficacy and tolerability of TDF/FTC and LPR/r for PBC patients on maintenance ursodeoxycholic acid therapy.

The trial was constructed as a double-blind randomized controlled crossover study of a 6-month duration using standard dose TDF/FTC 300/200 mg and LPR/r 440/100 mg BID versus placebo in PBC patients treated with standard UDCA (http://www.clinicaltrials.gov/ct2/show/NCT01614405). The study had limited enrollment because the majority of patients with PBC were unable to tolerate LPR/r. Indeed, over two thirds of patients discontinued LPR/r due to nausea, vomiting, diarrhea, abdominal pain, weight loss, and/or inability to swallow the tablets. The frequency of experiencing the gastrointestinal side effects was two to three times higher than those reported for patients with HIV. It remains to be resolved why this is the case. Indeed, abdominal complaints are not usually associated with PBC unless patients have concurrent celiac or inflammatory bowel disease. Therefore, pharmacological studies maybe warranted to investigate whether patients with PBC have a different ability to metabolize LPR/r because of ductopenia. Such studies may be important, as we have observed that some of the newer HIV protease inhibitors have superior antiviral activity to the betaretrovirus protease in vitro.

The preliminary 6-month data of placebo versus controls prior to crossover showed biochemical improvement from baseline with TDF/FTC and LPR/r therapy (Fig. 1), which was nearly double of previously observed in the AZT/3TC study [84]. However, the patients unable to tolerate the 6-month therapy were offered the opportunity to continue on TDF/FTC alone. All patients benefitting from therapy were then invited to enter an extended open-label phase of a further 18-month therapy to assess long-term efficacy and tolerability. While the long-term extension study is still ongoing, it is notable that patients able to continue with LPR/r have maintained a superior reduction in alkaline phosphatase levels than those on TDF/FTC alone. While we await the completion of the extended open-label study with collection of histological and clinical data, it is highly unlikely that LPRr treatment will be used for patients with PBC. In this regard, ongoing laboratory investigations indicate that other HIV protease and integrase antagonists have demonstrable activity against MMTV in vitro and in mouse models. The latter are being studied with view to finding superior combinations to treat patients with PBC in clinical trials.

## Future Studies to Determine a Causational Relationship Between Betaretrovirus and PBC

While a human betaretrovirus infection has been linked with PBC, a causal relationship has yet to be established. Several criteria have previously been suggested for proving causation, such as Koch’s postulates and Hill’s criteria [85]. While Koch’s postulates are difficult to obtain for complex diseases mediated by chronic infections on a specific genetic background, several of the postulates have been met in patients and in vitro (Table 1). Similarly, a few of the Bradford Hill criteria have been met, but better diagnostic assays will be required for large-scale epidemiological studies (Table 2).

**Table 1:** Use of Koch’s postulates to support a causal association of human betaretrovirus infection with PBC

1. The microorganism must be found in abundance in all organisms suffering from the disease, but should not be found in healthy animals.	Evidence for viral infection is found not only in ∼70 % of patient samples depending on the method used but also 5 to10 % of control subjects.
2. The microorganism must be isolated from a diseased organism and grown in pure culture.	Virus has been isolated in Hs578T cells co-cultured with peri-hepatic lymph node homogenates from PBC patients.
3. The cultured microorganism should cause disease when introduced into a healthy organism.	Virus induces the disease-specific phenotype in vitro with increased and aberrant PDC-E2 expression. MMTV is associated with a similar disease in mice. Mouse models with known genetic risk factors associated with PBC should be tested with the putative virus.
4. The microorganism must be re-isolated from the inoculated, diseased experimental host and identified as being identical to the original specific causative agent.	

Note that these postulates were originally created for acute bacterial infections with a high penetrance of disease. Koch’s postulates are too stringent to prove causal association with a prevalent agent in a chronic disease process, which is limited to susceptible individuals

**Table 2:** Support for a causal association of human betaretrovirus infection with PBC using Bradford Hill criteria

1. Strength	
A larger association is more likely to show causality.	Virus was detected in 70 % of PBC patient samples using immunohistochemistry, in situ hybridization, RT-PCR, and ligation-mediated PCR (to detect proviral integration) but only in small sample sets of <30 patients. Studies with a larger sample size are required to enhance causal strength.
2. Consistency	
Consistent findings strengthen the likelihood of causality.	Data should be independently confirmed by external sources.
3. Specificity	
The more specific an association between a factor and an effect is, the bigger the probability of causality.	Viral infection is not specific for PBC since evidence for viral infection is found in 5–10 % of control subjects. Infection may only cause disease in patients with specific genetic backgrounds. Nevertheless, antiretroviral therapy impacts on disease progression. Further studies are required to correlate virus levels and clinical improvement.
4. Temporality	
The effect has to occur following the cause.	Large epidemiological studies as well as the development of enhanced serological diagnostic assays are required.
5. Biological gradient	
Greater exposure should generally lead to greater incidence of the effect.	Large epidemiological studies as well as better understanding of MMTV zoonosis are still required.
6. Plausibility	
A plausible mechanism between cause and effect should be proposed.	In vitro experiments show that the disease-specific phenotype with AMA reactivity is enhanced in biliary epithelial cells following infection with the virus and may subsequently cause loss of tolerance.
7. Coherence	
Coherence between epidemiological and laboratory findings increases the likelihood of a causal relationship.	Marked female preponderance of PBC might be due to expression of female hormones that stimulate betaretrovirus long terminal repeat and increase viral replication.
8. Experiment	
Experimental evidence will increase the possibility of causality,	Purified virus was found to trigger disease-specific phenotype in vitro. Furthermore, antiretroviral therapy reduced disease progression in mouse models and in patients.
9. Analogy	
The effect of similar factors may be considered.	Similar viruses that cause cholangitis and autoantibodies have not been identified.

Note that the Bradford Hill criteria have insufficient applicability to prove causal association with a prevalent microbial infection and a chronic disease linked with a strong genetic component; large epidemiological studies using diagnostic tests with near 100 % sensitivity for both the genetic and microbial factors would be required

Studies in mice to further determine the role of MMTV in triggering cholangitis and anti-mitochondrial antibody production could enhance our understanding of the role of betaretrovirus infection in PBC. For example, while TDF/FTC and LPR/r were found to attenuate MMTV replication and liver disease in the NOD.c3c4 model [71], these inhibitors may target other retroviruses in humans and mice. Further studies on the NOD.c3c4 model are required that specifically inhibit MMTV replication, for example, through the use of MMTV-specific shRNAs and neutralizing anti-MMTV antibodies. Additionally, cross-fostering experiments could be performed on NOD.c3c4 offspring using mothers of different backgrounds that are MMTV free to determine whether cholangitis development in the NOD.c3c4 model is mediated by exogenous MMTV.

Sequencing of exogenous MMTV from NOD.c3c4 milk should help to characterize the specific replicative strain of MMTV, which would help to further implicate the virus in the disease process. For example, the MMTV-specific superantigen stimulates a cognate Vβ population of T cells, and if this subset is found in the liver, the observation can directly link the virus with the immune response [43]. Interestingly, studies with NOD mice have shown that Vβ3+ T cells are localized to the pancreas early in the disease, implicating MMTV in disease [86, 87]. This is somewhat surprising because the majority of Vβ3+ T cells should have been deleted by endogenous *mtv-3* superantigen. Since NOD.c3c4 mice encode *mtv-3* [9], identifying the T cell repertoire in the liver and resident lymph nodes at different stages of the disease could potentially link viral infection with the Vβ3+ T cells in the development of cholangitis.

## Prospectus

Similar to the role of MMTV in human breast cancer development, the association of a human betaretrovirus with PBC remains controversial. Further research is needed to clearly implicate betaretroviral infection in mouse models and in patients. Better serological and quantifiable nucleic acid tests will be required to perform large epidemiological studies and monitor virological response to antiretroviral treatment. The viral hypothesis of PBC will likely receive traction once clinical improvement can be directly associated with viral inhibition. Studies such as these can directly follow the lead of Barry Marshall who convinced his critiques that *H. pylori* caused peptic ulcer disease by using the therapeutic approach of eradicating bacterial infection and curing disease.
